# SE-MSLC: Semantic Entropy-Driven Keyword Analysis and Multi-Stage Logical Combination Recall for Search Engine

**DOI:** 10.3390/e27090961

**Published:** 2025-09-16

**Authors:** Haihua Lu, Liang Yu, Yantao He, Liwei Tian

**Affiliations:** 1 School of Computer Science, Guangdong University of Science and Technology, Dongguan 523083, China; luhaihua@pku.edu.cn (H.L.); heyantao@gdust.edu.cn (Y.H.);; 2 Guangdong AIoT Application Innovation Joint Laboratory, Guangdong University of Science and Technology, Dongguan 523083, China; 3 AIoT Edge Computing Engineering Technology Research Center of Dongguan City, Guangdong University of Science and Technology, Dongguan 523083, China

**Keywords:** search engine, semantic entropy, keyword importance analysis, multi-stage recall, logical combination recall

## Abstract

Information retrieval serves as a critical methodology for accurately and efficiently obtaining the required information from massive amounts of data. In this paper, we propose an information retrieval framework (SE-MSLC) that utilizes information theory to improve the retrieval effectiveness of inverted index retrieval, thus achieving higher-quality retrieval results in intelligent vertical domain search engines. First, we propose a semantic entropy-driven keyword importance analysis method (SE-KIA) in the query understanding module. This method combines search query logs, the corpus of the search engine, and the theory of semantic entropy, enabling the search engine to dynamically adjust the weights of query keywords, thereby improving its ability to recognize user intent. Then, we propose a hybrid recall strategy that combines a multi-stage strategy and a logical combination strategy (HRS-MSLC) in the recall module. It separately recalls the keywords obtained from the multi-granularity word segmentation of the query in the form of multi-queue recall and simultaneously considers the “AND” and “OR” logical relationships between the keywords. By systematically managing retrieval uncertainty and giving priority to the keywords with high information content, it achieves the best balance between the quantity of the retrieval results and the relevance of the retrieval results to the query. Finally, we experimentally evaluate our methods using the Hit Rate@K and case analysis. Our results demonstrate that the proposed method improves the Hit Rate@1 by 7.3% and the Hit Rate@3 by 6.6% while effectively solving the bad cases in our vertical domain search engine.

## 1. Introduction

The information explosion on the Internet has made search engines vital for knowledge access. While early search systems relied on basic keyword matching, advances in big data and artificial intelligence technology have raised user expectations towards deep query understanding and precise results. This evolution is further accelerated by the rise of retrieval-augmented generation (RAG) systems, where the quality of the initial retrieval phase critically determines the quality of the generated output [1,2].

The core goal of the search engine is to quickly and accurately return the most relevant results to the user’s query in the massive data. The effectiveness of a search engine hinges on its two-stage architecture: retrieval and ranking. The retrieval stage is responsible for screening out potentially relevant candidate sets from a large collection of documents, while the ranking stage further evaluates the relevance of candidate sets in detail. Among them, retrieval effectiveness in the retrieval stage is crucial in the overall search engine, which determines how many truly relevant documents the system can cover. If key documents are omitted in the retrieval stage, even if the subsequent ranking algorithm is powerful, it will seriously affect the overall performance of the system. Therefore, improving retrieval effectiveness is a fundamental challenge in optimizing the effectiveness of search engines.

The main challenges in enhancing search retrieval effectiveness stem from two aspects: the query understanding module and the recall module. The query understanding module encompasses tasks such as query preprocessing, query rewriting, query segmentation, synonym extension, keyword importance analysis, and intention recognition. Among them, accurately analyzing the importance of query keywords is crucial for deeply understanding user intent, as it enables the search engine to efficiently retrieve highly relevant content from massive information sources. Therefore, it is of great significance to boost overall retrieval performance. Traditional methods for keyword importance analysis, such as TF-IDF [3], BM25 [4], and BM25F [5], rely primarily on statistical features. While these approaches consider term frequency and distribution, they ignore semantic relationships between keywords, limiting their ability to handle the semantic complexity of natural language queries. As a result, they often fall short in accurately assessing keyword importance in real-world knowledge search engines. Word embedding models based on deep learning neural networks, such as Word2Vec [6], GloVe [7], and Qwen3 Embedding [8], can capture rich semantic relationships and similarities between words. These networks have been successfully applied to the importance analysis of query keywordsand have contributed to improved retrieval effectiveness. However, these solutions still suffered from several limitations in our practical application: (i) The complex network structure results in long inference times, which impacts search performance. (ii) They require a large amount of training data. (iii) They demand significant computational resources for both training and inference. A more recent context-aware term-weighting approach [9], which leverages a large pre-trained language model and the complete context of the query or document, dynamically assigns an importance weight to each term within it. While it achieves superior effectiveness by capturing deeper contextual semantics, its model structure is vastly more complex, leading to even greater online latency. To overcome these layered challenges that form the statistical shortcomings of traditional methods to the semantic yet inefficient nature of neural approaches, we propose a new query keyword importance analysis method, which is called semantic entropy-driven keyword importance analysis(SE-KIA). Our approach is designed to bridge this gap; it captures meaningful semantic relationships without relying on computationally intensive deep neural networks during inference. By leveraging lightweight semantic entropy measures derived from domain-specific query logs and corpus co-occurrence statistics, SE-KIA enables the search engine to dynamically estimate keyword importance in a computationally efficient, explainable, and scalable manner. This makes it particularly suitable for vertical domain search engines where both semantic understanding and operational efficiency are critical.

In modern search engines, the recall module primarily utilizes technologies such as inverted index retrieval, vector retrieval, and graph retrieval. Vector retrieval leverages advanced deep learning models to transform various types of data, including texts and images, into vector representations [10,11]. By measuring similarity between vectors, it effectively captures semantic relationships and supports complex content matching. Graph retrieval is based on the constructed knowledge graphs or user behavior graphs. It mines potential associations by utilizing the relationships between nodes and edges, thus enabling the discovery of in-depth information associations based on relationships [12,13]. Inverted index retrieval is a classic and fundamental search method that organizes data in a unique and efficient manner, laying a solid foundation for rapid information retrieval. The core concept of the inverted index is to establish a mapping relationship between each term during document collection and the documents that contain that term [14,15]. This reverse indexing structure from terms to documents significantly enhances retrieval efficiency, especially when dealing with large-scale text data, where its advantages are particularly prominent. In this paper, we focus on optimizing inverted index retrieval and propose a hybrid recall strategy that integrates multi-stage and logical combination methods (HRS-MSLC), with the goal of enhancing the relevance of retrieval results.

In this paper, we focus on the retrieval stage of inverted index retrieval, aiming to improve retrieval effectiveness and thus optimize the retrieval effect of the entire search engine. First, we propose a new keyword importance analysis method driven by semantic entropy in the query understanding module, enabling the search engine to more accurately understand user intent. Then, we introduce a hybrid recall strategy that integrates the concepts of multi-stage and logical combinations in the recall module, enabling the search engine’s retrieval results to be more relevant to the query.

In summary, the contributions of this paper are as follows:

- We propose a keyword importance analysis method driven by semantic entropy(SE-KIA) to achieve term weighting. Taking the theory of semantic entropy as its theoretical foundation, it makes use of the user information in search query logs and combines it with the corpus of the search engine, providing a comprehensive context for understanding the importance of keywords in the domain related to the search engine. Based on the integration of these three aspects, SE-KIA enables the search engine to dynamically adjust the weights of query keywords. As a result, it significantly enhances the search engine’s ability to accurately recognize user intent, ensuring that the search results are more relevant and better meet the users’ information needs.
- We propose a hybrid recall strategy with multi-stage recall and logical combination recall(HRS-MSLC). In the form of multi-queue recall, we separately recall the keywords obtained from the multi-granularity word segmentation of the query and simultaneously consider the “AND” and “OR” logical relationships between the keywords. This strategy aims to overcome the limitations of traditional recall methods, which often find it difficult to strike an appropriate balance between retrieving a sufficient number of relevant documents and ensuring highly relevant recall results.
- An in-depth analysis of the application effect of SE-KIA and HRS-MSLC in our vertical domain search engine was conducted. The Hit Rate@1 is improved from 85.6% to 92.9% (a significant increase of 7.3%), and the Hit Rate@3 is improved from 87.5% to 94.1% (a significant increase of 6.6%). Meanwhile, the problems of recall documents only hitting a single keyword and keyword importance analysis judgment error have been effectively solved, which proves that they can effectively improve the search performance.

The rest of our paper is organized as follows: Section 2 reviews related work. Section 3 introduces the retrieval stage in a search engine in general. In Section 4, we introduce our keyword import analysis algorithm, SE-KIA. In Section 5, we introduce our recall strategy, HRS-MSLC. Then, the results of the experiment are presented in Section 6. Finally, the conclusions are given in Section 7.

## 2. Related Work

### 2.1. Keyword Importance Analysis in the Query Understanding Module

The importance analysis of search query keywords has always been one of the core research directions in the field of intelligent search, which plays a key role in improving search efficiency and accurately understanding user intention. Many scholars have carried out in-depth research from different perspectives, promoting the continuous development of this field.

Early methods for analyzing the importance of keywords were mainly based on statistical features. Jones K S introduced TF-IDF, which evaluates keyword importance by combining term frequency with inverse document frequency [3]. Later, Robertson S E enhanced this approach with the BM25 algorithm, incorporating document length normalization and term frequency saturation [4]. While these statistical methods provide efficient quantitative measures, they overlook semantic relationships between keywords, limiting their accuracy in real-world search applications.

With the development of natural language processing technology, research based on semantic analysis has opened up a new approach for keyword importance analysis [16]. Semantic search is dedicated to accurately grasping the real intent of users through the words themselves in the user input. When multiple words frequently co-exist in the same document, they can be considered semantically related, addressing the limitations of statistical methods. However, challenges remain in handling complex and domain-specific semantics.

The rise of word embedding technology has brought new changes to keyword importance analysis. Word embedding models, such as Word2Vec [6], GloVe [7], etc., can map words to low-dimensional vector spaces so that semantically similar words are close in the vector space. In this way, rich semantic relationships and similarities between words can be learned. However, the word embedding model requires a lot of data and computational resources during the training process, and its representation effect may not be good for some rare words or new words.

In recent years, research combining user behavior analysis has gradually attracted attention. Users’ search logs, click behavior, and other data contain rich information; by digging through these data, we can understand the user’s usage habits and preferences for different keywords, which helps us better analyze the importance of keywords in queries and understand users’ search intentions [17,18].

Therefore, we propose a new keyword importance analysis method, called SE-KIA, which uses the user search query logs to mine information, combines the corpus of the vertical domain search engine and semantic entropy theory, and realizes the dynamic adjustment of online query keyword weights in the search engine, so as to better understand the user’s search intention.

### 2.2. Recall Module

Over the years, search engines have undergone significant development. They have gradually evolved from simple text matching-based retrieval tools in the early days to complex systems that integrate multiple advanced technologies, making them capable of precisely understanding user intentions and providing high-quality results. In this development process, technologies such as inverted index retrieval, vector retrieval, and graph retrieval in the recall module have played a crucial role.

The inverted index, a cornerstone of search technology, has evolved significantly since its inception. Inverted index retrieval takes terms as the core of the index. Its basic principle is to establish a mapping relationship from terms to the documents that contain them. During querying, relevant documents are quickly located based on this index. In the early days, the inverted index was mainly based on term matching, quickly screening out documents containing query terms from a vast number of documents. Later, as the explosive growth in data scale, it overcame many challenges in terms of storage and retrieval efficiency through optimization measures such as improving the dynamic update mechanism [19,20] and compression algorithms [15,21]. Despite the continuous emergence of new retrieval technologies, inverted index retrieval, due to its irreplaceable advantages, remains the fundamental basis of search engines.

Vector retrieval is a retrieval technology that emerged with the rise of deep learning. It maps queries and documents into a vector space and performs retrieval by calculating the similarity between embedding vectors. Huang, P.S. et al. proposed the deep semantic similarity model (DSSM), which uses a dual-tower neural network to encode texts into low-dimensional vectors for cosine similarity scoring [22]. Covington, P. et al. proposed a two-stage neural retrieval system to learn user and video embeddings for personalized recommendations [23]. Huang, J.T. et al. further developed a unified embedding framework for semantic search, integrating vector retrieval with traditional inverted indexes [24]. Scheerer, J.L. et al. accelerated multi-vector retrieval via dynamic similarity imputation and compressed scoring [25].

Graph retrieval is another important type of retrieval method that has emerged in recent years. Its core idea is to model the entities in the search engine, such as queries, documents, users, etc., and their relationships as a graph structure, and it uses graph algorithms to mine deep-seated association patterns. Classic algorithms, such as Deepwalk [26], node2vec [27], and DeepHub [28], generated node sequences through random walks, and then used word embedding technology to learn the representation of nodes. These methods are computationally efficient but have difficulty in capturing complex semantic information in the graph. The main challenges faced by graph retrieval are high computational complexity and difficulty in real-time updates.

Despite advancements in vector and graph retrieval technologies, inverted indexes remain fundamental to modern search engines owing to their superior engineering efficiency and result interpretability. This highlights the significance of optimizing the inverted index architecture for modern information retrieval systems.

## 3. Retrieval Stage in Search Engine

A search engine is typically divided into a retrieval stage and a ranking stage. The retrieval stage mainly aims to efficiently identify a candidate set relevant to the user’s query from a vast collection of documents or data. It is like a “coarse-screening” process, with the goal of finding all potentially relevant information as comprehensively as possible to lay the foundation for subsequent precise ranking and filtering. The ranking stage, on the other hand, precisely ranks the recalled candidate documents. By comprehensively considering factors such as the relevance between the documents and the query, the quality of the documents, and user preferences, it calculates the score of each document, ranks the documents that best meet the user’s needs at the top, and presents them to the user.

**Figure 1:**
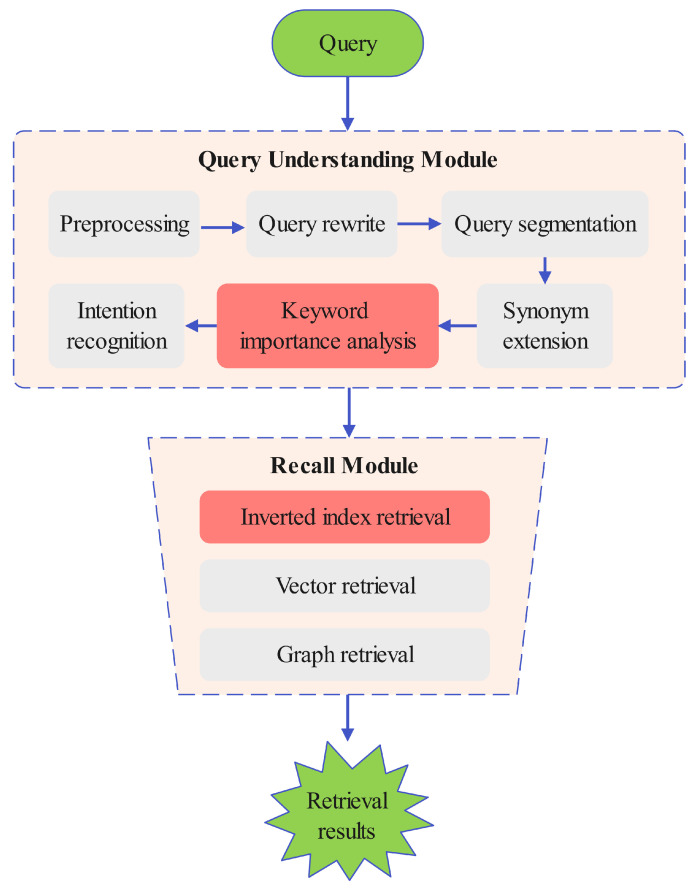
Retrieval stage architecture. The red box highlights the key module introduced in this work, while gray boxes represent existing components for context.

In this paper, we focus on the retrieval stage, which is a crucial stage within the entire search engine. Our goal is to improve the retrieval effectiveness of the retrieval stage, reducing the omission of valuable information during the initial screening process. In this way, the performance of the entire search engine can be enhanced, and the users’ search experience can be significantly enriched. The retrieval stage architecture in a search engine is described in Figure 1, which primarily consists of two core modules: the query understanding module and the recall module.

### 3.1. Query Understanding Module

The query understanding module is responsible for analyzing the query content input by the user and deeply interpreting the user’s search intention. The query understanding module performs complex processing on the user’s query through the following key components:

- Query preprocessing: It mainly performs the following preprocessing on queries to facilitate subsequent analysis by other modules, including removing useless symbols, uniformly converting cases, truncating extremely long queries, etc.
- Query rewrite: It optimizes user queries through two key techniques: query correction to fix spelling and syntax errors [29,30] and query completion to predict and supplements partial queries for better intent matching [31].
- Query segmentation: It divides the user query into meaningful words or phrases, which helps the search engine accurately understand the key points of user queries [32]. After word segmentation, the query terms can be precisely matched with the words in the knowledge base index, allowing for the rapid location of relevant documents or information.
- Synonym expansion: Users have different expression habits. Through synonym expansion, words that are synonymous or nearly synonymous with the query term can be included in the retrieval scope. In addition, in some domains, the words used by users may not be the typical words that best express their needs [33]. Synonym expansion is helpful in uncovering users’ potential true intentions.
- Keyword importance analysis: By analyzing the importance of query keywords to achieve precise term weighting, the search engine can quickly grasp the core of the query and produce the optimal retrieval output [9]. This is the key to having an in-depth understanding of users’ query intentions and is of great significance to improve the accuracy of search recall.
- Intention recognition: Through the accurate identification of users’ intentions, the search engine can provide results that meet expectations, thereby enhancing user satisfaction [34].

Among these components, keyword importance analysis plays a crucial role. In this paper, we focus on this component and propose a new method for analyzing the importance of keywords driven by semantic entropy.

### 3.2. Recall Module

In terms of the retrieval module, technologies such as inverted index retrieval, vector retrieval, and graph retrieval are widely used in modern retrieval systems.

- Inverted index retrieval: The inverted index is the most classic and crucial retrieval technology in search engines, enabling efficient retrieval of massive amounts of data. Its core idea is to construct a reverse index from terms to the documents containing these terms in the corpus. First, the document set in the corpus undergoes preprocessing, and a dictionary of all unique terms is built. Then, each term corresponds to an inverted list that records the IDs of the documents containing the term, along with additional information such as term frequency and position. When users input a query, the search engine segments it into keywords, retrieves relevant document IDs using the inverted index, and computes the final results through set operations. Currently, there are multiple open-source search engines, such as Solr [35] and Elasticsearch [36], which have built-in functions for efficiently constructing and querying inverted indexes.
- Vector retrieval: A typical vector retrieval system consists of two stages: offline processing and online retrieval. In the offline stage, documents are encoded into embeddings using pretrained models, then indexed via the approximate nearest neighbor(ANN) [37] algorithm for efficient similarity search. During online retrieval, queries are vectorized and matched against the index, with the top N results returned by similarity ranking.
- Graph retrieval: Graph retrieval is a technology for information retrieval. It is based on the graph data structure, with documents and entities as nodes and their relationships as edges. When a user initiates a retrieval request, the input keywords are converted into query conditions. The system will start from the nodes related to the query and conduct a traversal exploration along the edges according to the characteristics of the graph structure. Then, according to a certain sorting algorithm that comprehensively considers factors like relevance and importance, the results that best meet the user’s needs are presented to the user, thereby achieving efficient and accurate information retrieval.

In this paper, we focus on the optimization of inverted index retrieval and propose a hybrid recall strategy that integrates multi-stage recall and logical combination recall.

## 4. SE-KIA: Semantic Entropy-Driven Keyword Importance Analysis

In this section, we propose a keyword importance analysis method to achieve term weighting for the query understanding module in a search engine. We have a search engine in the vertical field of telecommunications, which contains a large number of knowledge documents, with the aim of helping users efficiently obtain knowledge. Previously, our search engine analyzed the importance of query keywords by assigning a static word weight to each term based solely on part-of-speech analysis. Such a simple keyword importance analysis method has the problem that term weight assignments are not accurate enough. Furthermore, a single global keyword importance table was used, causing the weight of each keyword to remain constant across all queries, thereby preventing the dynamic adjustment of keyword weights according to the query context. In fact, dynamic weighting is very important in search engines to distinguish the importance of the same keyword in different queries. For example, for the query “nr 7:3 timeslot ratio”, it can be said that the user clearly intends to find knowledge related to the timeslot ratio of 7:3. However, since the part of speech of the core term “7:3” is a number and the weight of the number of words is too low, the top-ranked results only recalled articles containing the keyword “timeslot ratio” rather than both “7:3” and “timeslot ratio”, resulting in inaccurate search results.

To address this issue, we explored multiple solutions. Initially, we attempted syntactic analysis but found that standard syntactic parsing typically assumes a sentence has only one root word, whereas search queries often contain multiple root words. Moreover, our analysis of search engine logs showed that 85% of user queries were keyword-based rather than complete sentences. As these fragmented queries generally violate fundamental syntactic structures, conventional parsing methods become inapplicable. Subsequently, we investigated utilizing traditional NLP models, such as BERT [38] and BiLSTM-CRF [39], to quantify keyword importance in queries. However, the implementation of this methodology also encountered multiple difficulties. First, data annotation is very difficult as humans cannot simply and directly access the importance of each keyword and must search the corpus to determine it. Second, the classification task lacked clear boundaries, resulting in unsatisfactory model performance during experimental validation.

However, implementing these approaches revealed a key insight: the importance of query keywords is closely related to the corpus distribution. This suggests that leveraging the existing corpus to automatically determine keyword importance would be more effective. Therefore, we propose a semantic entropy-driven keyword importance analysis (SE-KIA) method, which associates search query logs, the search engine corpus, and semantic entropy. First, our method analyzes search query logs to identify user behavior patterns and extract domain-specific common keyword pairs. Next, with the help of the corpus, the posterior results of joint and separate searches of query keywords are obtained. This is because the corpus, a vital component of the retrieval system, encompasses extensive text data indexed and retrieved by the search engine, thereby offering a comprehensive context for comprehending the meanings and significance of words within the domain relevant to the search engine. Then, semantic entropy is applied to determine the relative importance of keywords in the keyword pairs. Finally, a co-occurrence keyword graph is constructed to dynamically weight query keywords online so as to improve the search engine’s ability to understand the users’ intention and improve the retrieval effectiveness. The SE-KIA method consists of both online and offline components, with their detailed implementations presented in Algorithms 1 and 2, respectively.

### 4.1. Semantic Entropy Theoretical Analysis

**Figure 2:**
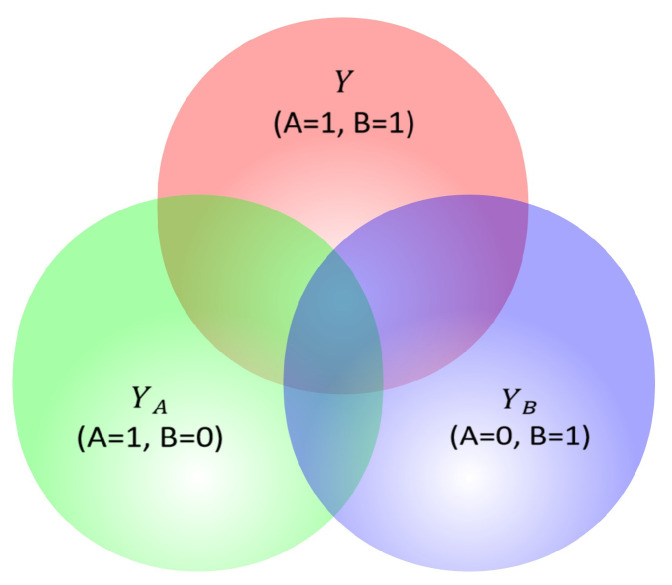
Venn diagram of posterior search results for a keyword pair.

Consider a keyword pair (A, B). As illustrated in Figure 2, the red circle represents posterior search results *Y* when A and B are jointly queried; the green circle represents the posterior search results $Y_{A}$ when only A is queried; and the blue circle represents the posterior search results $Y_{B}$ when only B is queried. The overlapping part of the red circle and green circle represents the intersection of *Y* and $Y_{A}$:

(1) $$Y|A=1,B=1\;\cap \;Y_{A}|A=1,B=0.$$

The overlapping part of the red circle and blue circle represents the intersection of *Y* and $Y_{B}$:

(2) $$Y|A=1,B=1\;\cap \;Y_{B}|A=0,B=1.$$

**Algorithm 1** SE-KIA (offline component)  1:**Input:** Query logs Q, search corpus C, co-occurrence threshold τ=10, search method Search(keywords, C) that returns an ordered list of documents ranked by relevance  2:**Output:** Keyword co-occurrence word graph G=(V,E)  3:**Step 1: Data Extraction**  4:Parse Q to extract all queries containing exactly two keywords  5:Filter out pairs with frequency f(A,B)>=τ, obtaining set P={(A,B)}  6:**Step 2: Posteriori Search Result Retrieval**  7:**for** each (A,B)∈P **do**  8:   Y←Search(A&B,C)  9:   $Y_{A}\leftarrow \mathrm{Search}\left(A,C\right)$10:   $Y_{B}\leftarrow \mathrm{Search}\left(B,C\right)$11:**end for**12:**Step 3: Semantic Entropy Calculation**13:**for** each (A,B)∈P **do**14:   Compute intersections:15:   $Y\;\cap \;Y_{A}=\left\{Z_{A1},Z_{A2},\ldots ,Z_{An}\right\}$16:   $Y\;\cap \;Y_{B}=\left\{Z_{B1},Z_{B2},\ldots ,Z_{Bm}\right\}$17:   Calculate occurrence counts:18:   $t_{A}\left(z\right)\leftarrow$ number of documents $z\in Z_{A}$ in $Y_{A}$19:   $t_{B}\left(z\right)\leftarrow$ number of documents $z\in Z_{B}$ in $Y_{B}$20:   Compute associated probabilities:21:   $p_{Y_{A}|Y}\left(z\right)=\frac{t_{A}\left(z\right)}{\sum_{z\in Z_{A}}t_{A}\left(z\right)}$22:   $p_{Y_{B}|Y}\left(z\right)=\frac{t_{B}\left(z\right)}{\sum_{z\in Z_{B}}t_{B}\left(z\right)}$23:   Compute semantic entropy:24:   $S\left(Y_{A},Y\right)=-\sum_{z\in Z_{A}}p_{Y_{A}|Y}\left(z\right)logp_{Y_{A}|Y}\left(z\right)$25:   $S\left(Y_{B},Y\right)=-\sum_{z\in Z_{B}}p_{Y_{B}|Y}\left(z\right)logp_{Y_{B}|Y}\left(z\right)$26:**end for**27:**Step 4: Relative importance determination**28:**for** each (A,B)∈P **do**29:   **if** $S\left(Y_{A},Y\right)<S\left(Y_{B},Y\right)$ **then**30:      $w_{AB}\leftarrow 1$31:  
 **else if** $S\left(Y_{A},Y\right)>S\left(Y_{B},Y\right)$ **then**32:      $w_{AB}\leftarrow -1$33:   **else**34:      $w_{AB}\leftarrow 0$35:   **end if**36:**end for**37:**Step 5: Graph Construction**38:Initialize G=(V,E) where V←∅, E←∅39:**for** each (A,B)∈P **do**40:   V←V∪{A,B}41:   $E\leftarrow E\cup \{\left(A,B,w_{AB}\right)\}$42:**end for**43:**return** G=(V,E)

**Algorithm 2** SE-KIA (online component)
  1:**Input:** User query $q=\{k_{1},k_{2},\ldots ,k_{n}\}$, keyword co-occurrence word graph G=(V,E), weight factor ▵weight=2, POS weight table W  2:**Output:** Keyword weights $\left\{w_{1},w_{2},\ldots ,w_{n}\right\}$  3:
**Step 1: Static Weight Assignment**
  4:**for** each keyword $k_{i}\in q$ **do**  5:   Retrieve part-of-speech tag $\mathrm{POS}(k_{i})$  6:   Assign static weight: $w_{i}\leftarrow W\left[\mathrm{POS}\left(k_{i}\right)\right]$  7:
**end for**
  8:
**Step 2: Dynamic Weight Adjustment**
  9:Generate candidate pairs: $P\leftarrow \{\left(k_{i},k_{j}\right)|1\leq i<j\leq n\}$10:**for** each pair $(k_{i},k_{j})\in P$ **do**11:   **if** $\left(k_{i},k_{j}\right)$ exists in *G* **then**12:      Retrieve precomputed relative importance $w_{ij}\leftarrow G.\mathrm{get}\_\mathrm{importance}\left(k_{i},k_{j}\right)$13:      **if** $w_{ij}=1$ and $w_{i}<w_{j}$ **then**14:          $w_{i}=w_{j}+▵weight$15:      **else if** $w_{ij}=-1$ and $w_{i}>w_{j}$ **then**16:          $w_{j}=w_{i}+▵weight$17:      **end if**18:   **end if**19:
**end for**
20:
**Step 3: New Queries Logging**
21:Insert *q* into log database L22:Periodically (e.g., daily): extract new pairs $\tilde{P}$ from L with frequency $f_{ij}\geq \tau$23:Update *G* with $\tilde{P}$24:**return** $\left\{w_{1},w_{2},\ldots ,w_{n}\right\}$


Here, we define the associated probabilities. Suppose $Y=\{y_{1},y_{2},\ldots ,y_{k}\}$, $Y_{A}=\left\{a_{1},a_{2},\ldots ,a_{g}\right\}$, and $Y_{B}=\left\{b_{1},b_{2},\ldots ,b_{l}\right\}$. Let $Y\;\cap \;Y_{A}=\left\{Z_{A1},Z_{A2},\ldots ,Z_{An}\right\}$, where *n* is the number of elements at the intersection of *Y* and $Y_{A}$. Let $Y\;\cap \;Y_{B}=\left\{Z_{B1},Z_{B2},\ldots ,Z_{Bm}\right\}$, where *m* is the number of elements at the intersection of *Y* and $Y_{B}$.

For the sets *Y* and $Y_{A}$, we define the probability that element $z\in Z_{A}$ occurs in set $Y_{A}$ as

(3) $$p_{Y_{A}|Y}\left(z\right)=\frac{t_{A}\left(z\right)}{\sum_{z\in Z_{A}}t_{A}\left(z\right)}.$$

where $t_{A}\left(z\right)$ is the number of occurrences of element $z\in Z_{A}$ in set $Y_{A}$. Since multiple distinct documents in the corpus may share the same title or correspond to the same semantic topic (e.g., different versions of an article), $t_{A}\left(z\right)$ counts all such documents collectively. Similarly, for the sets *Y* and $Y_{B}$, we define the probability that element $z\in Z_{B}$ occurs in set $Y_{B}$ as

(4) $$p_{Y_{B}|Y}\left(z\right)=\frac{t_{B}\left(z\right)}{\sum_{z\in Z_{B}}t_{B}\left(z\right)}.$$

where $t_{B}\left(z\right)$ is the number of occurrences of element $z\in Z_{B}$ in set $Y_{B}$.

Now, we construct the similarity measurement formula based on semantic entropy. The similarity between sets $Y_{A}$ and *Y* is determined as follows:

(5) $$S\left(Y_{A},Y\right)=-\sum_{z\in Z_{A}}p_{Y_{A}|Y}\left(z\right)logp_{Y_{A}|Y}\left(z\right).$$

The similarity between set $Y_{B}$ and *Y* is calculated as follows:

(6) $$S\left(Y_{B},Y\right)=-\sum_{z\in Z_{B}}p_{Y_{B}|Y}\left(z\right)logp_{Y_{B}|Y}\left(z\right).$$

Then, we take $S(Y_{A},Y)$ as the semantic entropy of keyword A and $S(Y_{B},Y)$ as the semantic entropy of keyword B. In this way, we can measure the relative importance of keywords A and B according to the semantic entropy. Concretely, the smaller the semantic entropy, the higher the relative importance of keywords.

Based on this, when analyzing keyword importance in the search engine, we can dynamically adjust the weight of keywords according to their relative importance. Specifically, when a keyword in a pair is deemed more important but has a lower weight than its counterpart, its weight will be dynamically adjusted to the sum of the counterpart’s static weight and an additional weight factor ▵weight. This mechanism enhances the keyword’s contribution to the overall query. For example, if A has higher importance but a lower weight than B, its weight will be increased as follows:

(7) weight(A)=weight(B)+▵weight.

Similarly, if B has higher importance but a lower weight than A, its weight will be increased as follows:

(8) weight(B)=weight(A)+▵weight.

In this way, we can dynamically adjust the weights of keywords according to the context information in the query. This makes the assignment of keyword weights more accurate and improves search retrieval effectiveness.

### 4.2. SE-KIA Architecture

**Figure 3:**
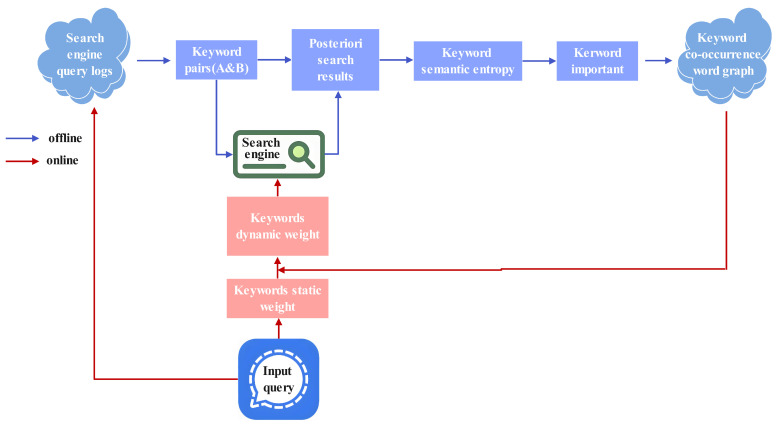
SE-KIA architecture.

As illustrated in Figure 3, the SE-KIA architecture comprises online and offline components.

The offline component constructs a keyword co-occurrence graph from search engine query logs, as described in Algorithm 1. The explanation of the steps are as follows:

Step 1: Data extraction: Search engine data, including user query logs and click-through behaviors, provides rich and valuable information. Especially for vertical search engines, mining user data helps us understand users’ usage habits and preferences regarding different keywords, enabling more accurate keyword importance analysis and intent understanding. To construct meaningful keyword pairs, we first extract a large volume of user query logs from our search engine database. Through statistical analysis of these logs, we make two key observations: (i) Queries composed of exactly two keywords account for a significant proportion of daily search traffic (approximately 68% in our dataset), indicating their practical relevance. (ii) For such two-keyword queries frequently submitted by users, the paired keywords tend to have strong semantic associations. They co-occur not only in user queries but also in the corpus with a significantly higher frequency than random keyword pairs. These observations motivate us to focus on frequent two-keyword queries, as they naturally form semantically coherent keyword pairs that align with real user search behaviors. In addition, longer queries (with three or more keywords) introduce higher combinatorial complexity and are often less frequent, leading to sparse and noisy data. Focusing on the most frequent two-keyword queries provides a robust and manageable set of keyword pairs for SE-KIA. Thus, we filter the query logs to retain only frequent queries consisting of exactly two keywords, using them as the basis for constructing our keyword pairs.

Step 2: Posteriori search result retrieval: For each keyword pair, we first retrieve the joint posteriori search results *Y* by querying both keywords concurrently against the search corpus. Subsequently, we obtain individual posteriori search results $Y_{A}$ and $Y_{B}$ by querying each keyword separately.

Step 3: Semantic entropy calculation: The semantic entropy of each keyword is computed based on the posterior search results. The semantic entropy of sets *Y* and $Y_{A}$ is computed by Equation (5), whereas the semantic entropy of sets *Y* and $Y_{B}$ is computed by Equation (6). A higher overlap in search results between sets *Y* and $Y_{A}$ or sets *Y* and $Y_{B}$ indicates lower semantic entropy (i.e., greater semantic certainty).

Step 4: Relative importance determination: We determine keyword relative importance through semantic entropy analysis. within each keyword pair, the keyword with lower semantic entropy is assigned higher relative importance.

Step 5: Graph construction: After extracting all keyword pairs and their relative importance, we construct a keyword co-occurrence word graph. This graph explicitly captures the importance relationships between co-occurring keywords in a query, enabling the search engine to dynamically adjust keyword weights during online query processing.

The online component is responsible for the dynamic keyword weighting process and uses the co-occurrence word graph during online query execution, as described in Algorithm 2. The explanation of the steps are as follows:

Step 1: Static weight assignment: When the users input a query, the search engine will first perform query preprocessing, query rewriting, query segmentation, and synonym extension. Then, our SE-KIA model first assigns a static initial weight to keywords based on their parts of speech. Words with different parts of speech will have different static weights. For example, for vertical search engines, some experts’ feedback business nouns will have a higher static weight.

Step 2: Dynamic weight adjustment: First, for each query, we generate all possible keyword pairs. For example, given query $Q=\{k_{1},k_{2},\ldots ,k_{n}\}$, we construct candidate pairs $P=\{\left(k_{i},k_{j}\right)|1\leq i<j\leq n\}$. Then, for each $(k_{i},k_{j})\in P$, we search for existence in the keyword co-occurrence graph, and then retrieve the precomputed relative importance if co-occurrence is confirmed. For the keyword with higher importance in each keyword pair, we dynamically increase its weight by Equation (7) or Equation (8), thereby increasing its importance in the overall query. If a keyword appears in multiple co-occurring pairs with consistently high relative importance, indicating its critical role in the query, we select the maximum static weight from all its paired keywords and add the accumulated weight factors to adjust its weight. In this way, dynamic weight adjustment based on semantic entropy is realized, and the search recall results are more accurate.

Step 3: New query logging: In our search engine, new user queries are logged into the log database in real time. This enables the regular extraction of novel keyword pairs from query logs, which are then incorporated into the keyword co-occurrence graph. Therefore, the coverage of the graph continues to expand to include more keyword pairs.

## 5. HRS-MSLC: Hybrid Recall Strategy with Multi-Stage and Logical Combination Recall

In this section, we present a hybrid recall strategy for the recall module, integrating both multi-stage and logical combination recall approaches. Currently, there are several open-source search engines that can help us efficiently build an inverted index search engine, such as Solr and Elasticsearch. Our vertical search engine employs Solr for its robust phrase query and boolean query capabilities and integrates our hybrid recall strategy, thus significantly enhancing the accuracy of retrieval.

### 5.1. Multi-Stage Recall Strategy

The query segmentation component in a search engine usually adopts the method of multi-granularity word segmentation, aiming to comprehensively analyze the users’ intention and ensure the quantity of retrieved results. First, obtaining phrases or words through coarse-grained segmentation can capture the complete semantics, conform to users’ precise expressions, and improve the relevance of retrieval. Then, the phrases may be further segmented into word granularity or words into subword granularity through fine-grained segmentation to cover more information, enrich the retrieved results, and ensure the quantity of retrieved items. Let a query *q* be segmented into the following multi-granularity terms:

(9) $$\begin{array}{cc} \; & Coarse-grained\;terms:P=\left\{p_{1},p_{2},\ldots ,p_{n}\right\}\left(phrase/words\right),\\ \; & Fine-gained\;terms:S=\left\{s_{1},s_{2},\ldots ,s_{n}\right\}\left(words/subwords\right). \end{array}$$

The traditional single-stage scoring function for document *d* is

(10) $${\mathrm{Score}}_{\mathrm{single}}\left(d\right)=\sum_{p\in P}w_{p}\cdot f\left(p,d\right)+\sum_{s\in S}w_{s}\cdot f\left(s,d\right).$$

where f(·,d) is a generalized correlation scoring function, with f(p,d) and f(s,d) assessing the semantic relevance of term *p* and term *s* in document *d*, respectively. $w_{p}$ and $w_{s}$ are term weights. It can be seen that this is a case of using phrases, words, and subwords simultaneously for retrieval. However, this will lead to a critical issue: high-frequency subwords may dominate the results, thereby suppressing higher-precision phrase and word matches and consequently compromising retrieval accuracy. For example, given the query “bts3203”, the segmentation component further decomposes it into the subwords “bts” and “3203”. The search engine then uses all three terms (the original query and subwords) concurrently to recall related documents. However, this approach creates a scoring imbalance: documents containing only the high-frequency subword “bts” receive inflated relevance scores and dominate the top results, while documents containing the exact term “bts3203”, although more semantically relevant, are deprioritized or even squeezed out of the recall results due to their lower scores, ultimately degrading retrieval precision.

Therefore, we propose a multi-stage recall strategy, replacing the original single-stage approach that combined phrases, words, and subwords with a two-stage recall approach. Specifically, suppose we need to retrieve the K documents that are the most relevant:

- Stage 1: Phrase-priority retrieval: Keywords derived from coarse-grained word segmentation are used for initial retrieval.
  (11) $$D_{1}=Top-K\left\{d|Score_{1}\left(d\right)=\sum_{p\in P}w_{p}\cdot f\left(p,d\right)\right\}.$$
- Stage 2: Subword-controlled expansion ($if\;D_{1}<K$): Keywords derived from fine-grained word segmentation are applied for supplementary retrieval.
  (12) $$D_{2}=Top-K\left\{d|Score_{2}\left(d\right)=\beta \sum_{s\in S}w_{s}\cdot f\left(s,d\right)\right\}.$$
  To prevent the supplementary retrieval stage from over-representing the impact of subword matches, which are inherently more numerous but potentially less meaningful, we introduce the damping factor β∈[0,1]. This factor controls the contribution of the subword-based retrieval score to the final ranked list.
- Final Results:
  (13) $$D_{final}=D_{1}\cup \left\{d\in D_{2}|Score_{2}\left(d\right)>\sigma \right\}.$$
  where σ is a fusion threshold. This hierarchical approach enhances retrieval effectiveness while mitigating the issue where subword matches disproportionately dominate coarse-grained term results.

In our implementation, we employ a multi-queue recall approach. The system processes both recall stages concurrently through separate queues, which are merged after completion. The primary queue (stage 1) results take precedence, while the secondary queue (stage 2) serves as a supplement when the initial recall yields insufficient results. This architecture reduces the time complexity from O(|P|+|S|) to O(max(|P|,|S|)), which reduces the time required for search retrieval. This improvement in time efficiency is achieved at the cost of minimal space overhead, with the space complexity increasing from O(K) to O(2K) for storing document IDs and titles. This represents a highly favorable trade-off in search engine design, where we leverage readily available memory to achieve lower retrieval time while simultaneously securing a substantial improvement in retrieval effectiveness.

### 5.2. Logical Combination Recall Strategy

In the traditional search recall process, only the “OR” relation among the query keywords is used for retrieval. This approach leads to two significant limitations: First, documents containing frequent single-query terms often receive inflated relevance scores and are ranked high despite their potentially low actual relevance to the query. Second, more relevant documents that contain multiple-query terms may be ranked lower due to lower scores. This mismatch between retrieval scoring and true document relevance significantly compromises search quality. For instance, when searching for “5G enables smart fisheries”, the top-ranked result might be a document like “5G Wireless Technology Evolution White Paper”. Although it is completely irrelevant to applications in smart fisheries, it receives a high score solely due to it containing numerous terms such as “5G”. This demonstrates how term–frequency bias can lead to fundamentally mismatched results.

To address these limitations, we propose a logical combination recall strategy that combines both “OR” and “AND” relations between query terms. Recognizing the inherent complexity of real-world search queries, our approach dynamically balances (1) precision-focused retrieval by applying strict “AND” logic to search documents containing all specified keywords, making it particularly valuable for targeted queries requiring exact matches by ensuring that only highly relevant results are returned and (2) recall-oriented retrieval by Employing “OR” logic to capture documents containing any query term, ensuring comprehensive coverage of potentially relevant results. This dual-mode strategy achieves an optimal trade-off between precision and recall.

Our logical combination recall strategy’s processing pipeline implements the following steps:

1. Selective “AND” constraints: From all query keywords sorted by weight, we select the top 5 highest-weighted keywords, and when the total keywords is less than 5, the top 80% of highest-weighted keywords are selected (rounded up). These selected keywords form mandatory “AND” conditions.
2. Comprehensive “OR” coverage: All query keywords (including the AND-selected ones) participate in “OR” matching.
3. Combined logical operation: The final recall condition expression for the Solr search engine is as follows:
  (14) $$\begin{array}{cc} condition= & (selected\_keyword_{1}\;AND\;selected\_keyword_{2}\;AND\;\ldots )\;OR\;\\ \; & (keyword_{1}\;OR\;keyword_{2}\;OR\;\ldots \;OR\;keyword_{n}). \end{array}$$

This balances the following: (1) Precise recall first: The“AND” operation strictly matches important keywords, with recall documents prioritizing the inclusion of all selected keywords. (2) Wide coverage guarantee: The “OR” operation maintains broad result coverage, with recall documents containing any keywords. Therefore, the search engine can prioritize recalling documents containing multiple query keywords, effectively addressing the problem that the retrieved documents only contain a single query keyword, making the recall results more accurate and highly relevant to the user’s query.

### 5.3. Hybrid Recall Strategy

**Figure 4:**
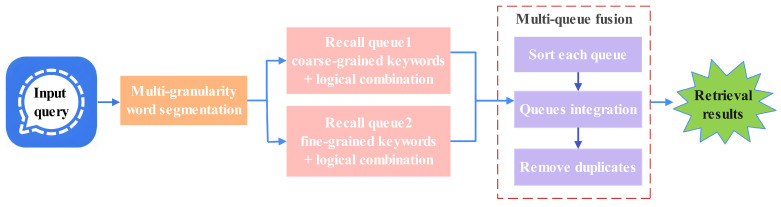
HRS-MSLC architecture.

To systematically integrate the advantages of both multi-stage and logical combination recall approaches, we propose a hybrid recall strategy with a multi-stage and logical combination (HRS-MSLC). The architecture of HRS-MSLC is shown in Figure 4, and its processing is as follows:

1. Word segmentation: The domain-specific segmentation component analyzes each query using multi-granularity tokenization, generating both coarse- and fine-grained keyword sets.
2. Parallel retrieval: The recall module employs two concurrent retrieval queues. Queue1 uses coarse-grained keywords, and queue2 processes fine-grained keywords. At the same time, in these two queues, we incorporate the logical combination strategy, adding two types of logic, “AND” and “OR”, between the keywords.
3. Multi-queue fusion: The system merges results from the two queues through a prioritized fusion strategy: (1) Each queue’s results are first ranked by their recall scores. (2) Queue1 results take precedence in the final output. (3) Queue2 results supplement when queue1 returns insufficient matches. (4) Duplicate results are removed to ensure unique results.

## 6. Experiments

### 6.1. Dataset

We evaluate our method on following two datasets.

#### 6.1.1. Vertical-UserTest

The Vertical-UserTest dataset comprises 600 real-world user queries sampled from our search engine logs in the communication domain. The corresponding corpus consists of professionally curated knowledge documents from the same vertical. All target documents were carefully annotated by domain experts to ensure high-quality and business-aware labels. Vertical-UserTest is used as the primary test dataset in our ablation studies, owing to the proposed method’s focus on vertical search engine optimization.

#### 6.1.2. TREC-CAR

To further assess the generalizability of our method, we also employ TREC-CAR (complex answer retrieval), a publicly available benchmark dataset introduced by Dietz et al. [40]. The corpus consists of all English Wikipedia paragraphs except the abstracts. Each query is constructed by concatenating a Wikipedia article title with the title of one of its sections. The target documents are the paragraphs within that section. We use the official test set from the TREC-CAR 2017 evaluation, which contains approximately 2250 queries.

### 6.2. Evaluation Method

To evaluate the effectiveness of our semantic entropy-driven keyword importance analysis (SE-KIA) method and hybrid recall strategy with a multi-stage and logical combination (HRS-MSLC), we employed two approaches to demonstrate their improvement on the quality of the retrieval results.

#### 6.2.1. Hit Rate@K

Hit Rate@K [41] evaluates the proportion of queries for which at least one relevant document is included in the top K retrieved candidates, which is widely used in the retrieval stage. In real-world search scenarios, users generally do not browse a large number of search results but tend to look at the first few results. This user behavior underscores the critical importance of optimizing the Hit Rate@K, ensuring that the most relevant documents appear in the top positions of the retrieved results. Evaluating the Hit Rate@K effectively simulates real user behavior, as it measures system performance specifically within the result range that users actually engage with. Even with high overall recall, poor top N performance leads to user dissatisfaction, since most users never discover relevant results ranked beyond the first few positions. Specifically, in our experiment, the Hit Rate@K is computed as

(15) $$HitRate@K=\frac{\sum_{q\in Q}I\left(d_{q}\in R_{q}^{\left(K\right)}\right)}{\left|Q\right|}.$$

where *Q* is the set of all queries in our test dataset, |Q| is the total number of queries, $d_{q}$ is the target documents of query *q* in the test dataset, $R_{q}^{\left(K\right)}$ is the top K retrieval results of query *q* returned by the search engine, and I(·) is an indicator function, which returns 1 if the condition is satisfied and 0 otherwise.

In practical search engines, Hit Rate@1 and Hit Rate@3 are critical metrics for evaluating retrieval effectiveness. The Hit Rate@1 measures whether the highest-ranked result of a query is consistent with the target documents, while the Hit Rate@3 assesses whether the target document is covered among the top three results. Therefore, we conducted comparative experiments to evaluate our method’s effectiveness: under identical test datasets and query conditions, the optimized system demonstrates superior retrieval effectiveness in critical top-ranked positions (top 1 and top 3) compared to the baseline version.

#### 6.2.2. Case Analysis

Case studies are of great significance in many fields [42,43]. Similarly, case analysis is a fundamental methodology for evaluating and enhancing search engine performance [44]. By systematically analyzing actual user queries and their corresponding retrieval results, we can uncover specific retrieval deficiencies, develop targeted algorithmic improvements, and validate optimization effectiveness through comparative experiments. In our experiments, by directly comparing the performance before and after the recall enhanced by the method we proposed, we can verify the effectiveness of retrieval optimization. We specifically select representative queries in our search engine, including known failure cases, to conduct detailed module-level analyses. These experiments reveal how our proposed method better captures users’ intent and returns more relevant results in critical top-ranking positions.

### 6.3. Results

In this section, we evaluate the effectiveness of optimizing SE-KIA and HR-MSLC, respectively, and their overall performance.

#### 6.3.1. SE-KIA Evaluation

The SE-KIA method operates by first identifying keyword pairs in the query through the offline keyword co-occurrence graph. For keywords with higher importance within these pairs, we dynamically adjust their weights based on the initial static weights and a weight factor. We determined the optimal weight factor ▵weight in Equations (7) and (8) through comparative experiments. The experimental results, as shown in Table 1, indicate that in our search engine and test set, the Hit Rate@1 and Hit Rate@3 are the highest when ▵weight is 0.2. This balance is crucial, as it optimally regulates the influence of the semantic entropy analysis result on final keyword weights. When ▵weight is set too low, the effect of dynamic weight adjustments is weakened, failing to emphasize core keywords in the query sufficiently. Conversely, an excessively high ▵weight value may cause the system to overly emphasize a single dominant keyword, disproportionately suppressing other terms and consequently losing vital query context. Through experimental validation, ▵weight=0.2 is confirmed as the optimal balance, which effectively enhances the discriminative power of core keywords while preserving meaningful contextual signals from other terms.

Furthermore, we evaluate the retrieval effectiveness of different keyword importance analysis methods within the BM25F framework. The BM25F algorithm is a cornerstone of modern information retrieval and is renowned for its effectiveness and robustness in scoring documents based on term frequency and field structure. Therefore, to establish a strong and realistic baseline, we first implemented Domain-BM25F, a retrieval system based on the BM25F algorithm that is enhanced by incorporating essential vertical domain optimizations, such as domain-specific tokenization and intent understanding. This optimized Domain-BM25F system serves as our foundational baseline, representing a competitively tuned traditional retrieval model. We than compare our proposed SE-KIA model against two methods: the standard Domain-BM25F algorithm and a static weight method. The static weight method enhances BM25F by incorporating fixed static weights to query keywords based on inverse document frequency (IDF) and part-of-speech features. The results are presented in Table 2. As can be seen, the static weight enhancement provides a measurable improvement over the standard BM25F algorithm. Furthermore, our SE-KIA model has further improved the Hit Rate@1 from 85.6% to 89.2% (an increase of 3.6%) and the Hit Rate@3 from 87.5% to 90.7% (an increase of 3.2%) relative to the static weight method. The results demonstrate that modifying the term-weighting method within the BM25F framework is highly effective and that our SE-KIA method significantly outperforms both the powerful BM25F baseline and the static weight method.

To validate the effectiveness of our SE-KIA method more intuitively, we conducted a comparative case analysis within our vertical search engine, evaluating three keyword weighting approaches: (1) No weight: This control group applies no weighting to query keywords, serving as a reference for minimum expected performance. (2) Static weight: The second control group assigns fixed static weights to query keywords based on corpus-wide inverse document frequency (IDF) and part-of-speech features. (3) Dynamic weight (SE-KIA): The third one is our dynamic weighting approach, SE-KIA, which dynamically adjusts the static weight of query keywords through the query context and the offline-constructed keyword co-occurrence graph. Experimental results demonstrate that our SE-KIA dynamic adjustment mechanism more accurately captures the actual importance of query keywords.

**Figure 5:**
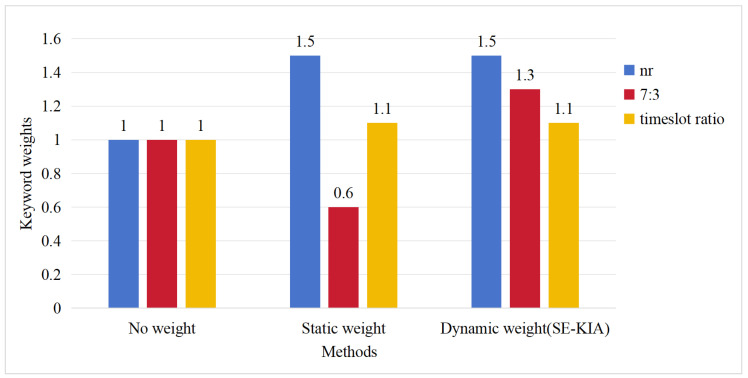
Keyword weights across analysis methods for the query “nr 7:3 timeslot ratio”.

For example, for the query “nr 7:3 timeslot ratio”, our search engine will first segment the query into three keywords: “nr”, “7:3”, and “timeslot ratio”. The complete keyword weight assignment results are illustrated in Figure 5: (1) No weight: When no weighting scheme is applied, all keywords in the query are assigned equal weights, and the default weight is one. This approach fails to capture their relative importance, significantly limiting the system’s ability to prioritize relevant search results. (2) Static weight: When using only static weighting, the assigned weights for the keywords “nr”, “7:3”, and “timeslot ratio” are 1.5, 0.6, and 1.1, respectively. This weighting scheme fails to accurately capture the user’s search intent. The core keyword “7:3” is clearly the central focus of the timeslot ratio query, but it is systematically assigned a lower weight (0.6) due to its part-of-speech classification as a numerical term, despite its potential significance in specific queries. The “timeslot ratio”, as a domain phrase, gains relatively high weights (1.1) by default, causing it to dominate the search results. This results in the importance of the keyword “7:3” being significantly lower than the keyword “timeslot ratio”. This weighting imbalance leads to the retrieval of documents ranked at the top that only contain the keyword “timeslot ratio” but without the critical “7:3” specification, fundamentally failing to meet the user’s search intent. (3) Dynamic weight (SE-KIA): Our SE-KIA method will further adjust the keyword static weights based on the query context. It will query the pre-constructed keyword co-occurrence graph and identify “7:3” and “timeslot ratio” as a co-occurrence pair, with “7:3” showing higher relative importance. To better reflect this importance, SE-KIA dynamically adjusts the weight of “7:3” upward to the static weight of “timeslot ratio” plus the weight factor while maintaining the weights of other keywords. Finally, the weights of the keywords “nr”, “7:3”, and “timeslot ratio” are 1.5, 1.3, and 1.1, respectively. It can be seen that the relative importance of “7:3” in the query is enhanced. This adjustment ensures that the top-ranked retrieval results contain both “7:3” and “timeslot ratio”, accurately reflecting the user’s search intent. Case analysis results demonstrate that our SE-KIA method significantly enhances query understanding by dynamically capturing contextual relationships between query keywords and identifying their relative importance, thus achieving more precise document retrieval.

To evaluate the practical overhead of the proposed SE-KIA model, we measured the average additional search time per query. The online computation of SE-KIA primarily involves querying a pre-computed keyword co-occurrence graph, a highly optimized operation that adds only 1.5 ms of latency on average. This overhead is negligible compared to the total search time of approximately 150 ms. Given the substantial improvement in retrieval effectiveness, the added latency becomes even more insignificant. Regarding resource utilization, the memory footprint of the online inference service is minimal; the large keyword co-occurrence graph is stored externally in a MongoDB database, which operates in an offline capacity. This architectural separation ensures that the online service remains lightweight and stateless, making the SE-KIA method highly efficient and suitable for large-scale, real-time online serving.

#### 6.3.2. HRS-MSLC Evaluation

**Figure 6:**
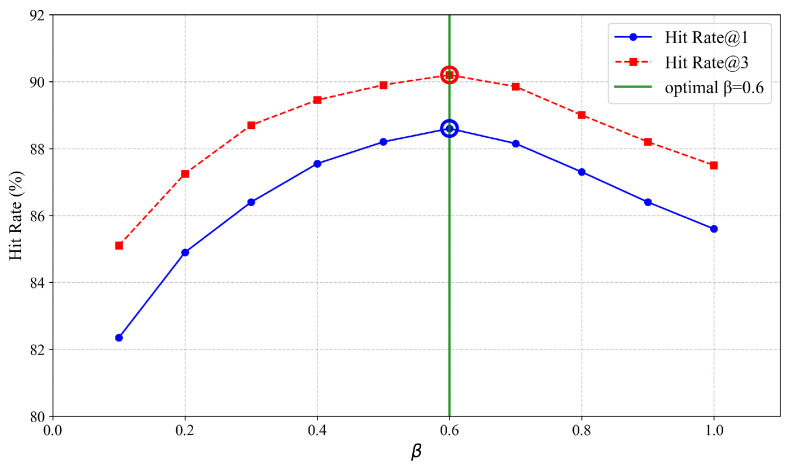
Impact of the parameter β on the hit rate.

Our HRS-MSLC model integrates two key components: a multi-stage recall strategy and a logical combination recall strategy. For the multi-stage recall strategy, we examined the impact of the damping factor β in Equation (12) on the overall retrieval performance by conducting a sensitivity analysis. The value of β is varied from 0.1 to 1.0. As illustrated in Figure 6, the performance metrics HitRate@1 and HitRate@3 initially increase as β rises from 0.1, reach an optimum around β=0.6, and then subsequently decrease. This trend validates the necessity of incorporating β: a β value that is too small unduly suppresses the beneficial effect of subword expansion, while a β value that is too large allows noisy subword matches to degrade the quality of the initial phrase-based retrieval. Through experimental validation, β=0.6 is established as the optimal value, effectively mitigating the issue where subword matches disproportionately dominate coarse-grained term results.

Furthermore, we conducted a comprehensive evaluation of HRS-MSLC through ablation experiments with the conventional OR-based strategy as a baseline, which uses only one queue for recall and connects all keywords with “OR” operators to retrieve documents containing any query term. This involved (i) independent evaluation of each component’s performance and (ii) analysis of their combined synergistic effects. The experimental results are shown in Table 3. The Hit Rate@1 and Hit Rate@3 of the original system are 85.6% and 87.5%. When we solely implement the multi-stage recall strategy in the original system, the Hit Rate@1 and the Hit Rate@3 are increased by 3% and 2.7%. Then, when we only use the logical combination recall strategy, the Hit Rate@1 and the Hit Rate@3 are increased by 2.5% and 2.2%. Finally, when we use both of these recall strategies simultaneously, that is, adopting the hybrid recall strategy, the Hit Rate@1 and the Hit Rate@3 are increased by 4.6% and 4.1%. The results demonstrate that both the multi-stage recall strategy and the logical combination recall strategy independently improve retrieval effectiveness. More significantly, the hybrid recall strategy achieves greater improvements, confirming the synergistic benefits of combining both approaches.

To validate the effectiveness of our SE-KIA method more intuitively, we conducted a comparative case analysis within our vertical search engine, evaluating two retrieval strategies in our vertical search engine. Unlike the OR-based method, our HR-MSLC method implements a dual-queue architecture: the first queue processes coarse-grained word segmentation, while the second employs fine-grained word segmentation, with both queues utilizing combined “AND”and “OR” operators for balanced accuracy and quantity of retrieval results. The system prioritizes results from the first queue and supplements with the second queue only when necessary to meet the recall quantity.

For example, for the query “AAU5613 installation guide”, our system first performs multi-granularity word segmentation, generating five keywords: “aau5613”, “aau”, “5613”, “installation”, and “guide”. Among them, the two keywords “aau” and “5613” are the subwords obtained by further segmenting the phrase “aau5613”. The OR-based method generates the following recall condition expression for the Solr search engine:

(16) $$\begin{array}{c} “aau5613”\;OR\;“aau”\;OR\;“5613”\;OR\;“installation”\;OR\;“guide”. \end{array}$$

This retrieval expression retrieves any document that contains at least one query keyword. In our corpus, the generic subword “aau” appears frequently across some documents, causing them to achieve high retrieval scores and dominate top results. Conversely, documents containing the specific target term “aau5613” rank lower or are excluded entirely due to insufficient scores, significantly compromising result accuracy. Our HR-MSLC method generates the following two recall condition expressions of two queues for the Solr search engine:

(17) $$\begin{array}{cc} Queue1: & \;(“aau5613”\;AND\;“installation”)\times w\;OR\;\\ \; & “aau5613”\;OR\;“installation”\;OR\;“guide”,\\ Queue2: & \;(“aau”\;AND\;“installation”\;AND\;“guide”)\times w\;OR\;\\ \; & “aau”\;OR\;“5613”\;OR\;“installation”\;OR\;“guide”. \end{array}$$

**Figure 7:**
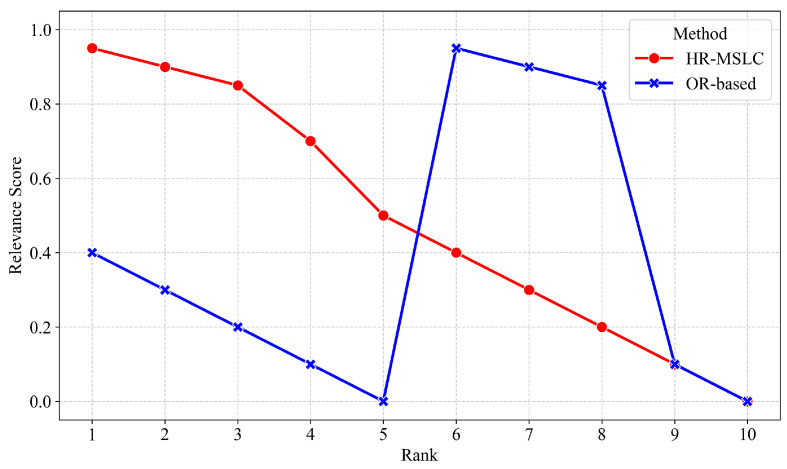
Relevance of the top 10 results across recall strategies for the query “AAU5613 installation guide”.

In the final retrieval results, since Queue1 has higher priority, documents containing the coarse-grained segmentation “aau5613” are ranked above those containing only the subword “aau” in the recall results, demonstrating the effectiveness of the multi-stage recall strategy. Additionally, the recall condition expression has a factor w for the AND-connected part, indicating that when documents match multiple AND-connected keywords, their recall score will be multiplied by the factor. Therefore, documents matching multiple AND-connected keywords simultaneously receive significantly higher recall scores and rank higher than those matching only a single keyword, improving the relevance of the top-ranked retrieved documents. This validates the effectiveness of the logical combination recall strategy. Figure 7 illustrates the relevance between the top 10 recall results and the target documents under different recall strategies. It can be observed that the OR-based method exhibited a significant sorting anomaly, where the document with the highest relevance score was mistakenly placed in the 6th to 8th positions, while the relevance of the top 5 results was not high. This is because this method provides higher scores to the documents when the subword “aau” appeared frequently, whereas our HR-MSLC model correctly prioritized precise phrase matching. It is evident that our HRS-MSLC model effectively addresses the problem of subword overshadowing and single-keyword matches, thereby enhancing retrieval effectiveness. This innovative design simultaneously ensures sufficient result quantity while significantly improving relevance, demonstrating clear advantages over baseline retrieval methods.

To evaluate the high efficiency of HRS-MSLC, we measured the reduction in average search time per query. The multi-stage recall strategy uses a parallel multi-queue retrieval mechanism, which significantly reduces time complexity through concurrent processing. Although the logical combination recall strategy introduces slight overhead in each queue, the system still achieves considerable performance improvement. Experimental results showed that the average search time per query is reduced by 8 ms. This improvement, however, involves a trade-off in memory usage. Since each queue retrieves the full anticipated number of documents, the total memory footprint during the recall stage increases. It is worth noting that the additional memory is primarily used to store document IDs and titles, resulting in only a minimal increase in memory consumption. This design reflects a typical space-for-time trade-off: devoting more memory to parallel queues enables substantially faster query processing, an essential benefit in low-latency, large-scale retrieval scenarios. Therefore, given that memory is a scalable resource and that the system achieves both a significant reduction in search time and a notable improvement in retrieval effectiveness, this trade-off is highly advantageous.

#### 6.3.3. Overall Evaluation

To thoroughly evaluate the performance gains from the ground up, we conducted ablation experiments using multiple baselines. The raw Domain-BM25F retrieval results serve as the foundational baseline, with Hit Rate@1 and Hit Rate@3 values of 78.2% and 80.4% respectively. This provides the benchmark for the value added by any subsequent system built upon it. Taking the static weight system as our main comparison point, we evaluated the overall performance improvements of SE-KIA and HRS-MSLC. The results are presented in Table 4. The Hit Rate@1 and Hit Rate@3 of the static weight system are 85.6% and 87.5%, which already represents a significant improvement over the Domain-BM25F baseline. When we solely implement the SE-KIA method in this system, the Hit Rate@1 and the Hit Rate@3 are further increased by 3.6% and 3.2%. Then, when we only use HRS-MSLC, the Hit Rate@1 and the Hit Rate@3 are increased by 4.6% and 4.1%. Finally, when we use both SE-KIA and HRS-MSLC simultaneously, the Hit Rate@1 and the Hit Rate@3 are increased by 7.3% and 6.6% relative to the the static weight system. The results demonstrate that our static weight system framework adds substantial value over the core BM25F retrieval model. Further, both SE-KIA and HRS-MSLC individually enhance retrieval effectiveness, with their combined application yielding even more significant improvements.

Finally, we conducted comparative experiments on the TREC-CAR dataset to evaluate the generalizability of our proposed method. For TREC-CAR, we use mean average precision (MAP). As shown in Table 5, the official reported MAP value for the BM25 baseline is 15.3. It can be observed that both our Domain-BM25F baseline and the static weight system significantly outperform this baseline. Moreover, our proposed method achieves further performance gains beyond both the enhanced Domain-BM25F baseline and the static weight system.

However, according to the experimental results, the improvement brought by our proposed method is more pronounced on the Vertical-UserTest dataset than on the TREC-CAR dataset. This can be attributed to the fact that our SE-KIA method, which relies on keyword co-occurrence pairs, was constructed using real user logs in a vertical domain, giving it strong domain-specific expertise. In contrast, TREC-CAR is built on an open Wikipedia corpus, where keyword co-occurrence pairs are far more numerous, more sparse, and less domain-focused. Furthermore, in the HRS-MSLC method, the multi-granularity tokenizer used in the multi-stage recall strategy has been optimized for the vertical domain. This optimization incorporates a large number of expert-annotated and crowdsourced domain-specific terms and phrases, an advantage that TREC-CAR does not possess. Notably, even within the vertical domain where baseline search performance is already strong, our approach demonstrates significant and measurable gains, highlighting its capability to push the boundaries of what is achievable in specialized search environments. Therefore, the proposed method is particularly suitable for vertical domain search engines, while further adaptation and optimization would be needed for general-purpose web search.

### 6.4. Discussion

The experimental results demonstrate that our SE-MSLC has outstanding performance and reveal four important findings:

(1) Traditional keyword importance analysis methods, such as TF-IDF or part-of-speech-based approaches, assign fixed static weights to keywords, failing to dynamically adjust keyword weights according to the context of the query. In contrast, our proposed SE-KIA method achieves superior adaptability that static methods cannot match. It can adjust the weights of keywords based on changes in the semantic context of the query, meaning that the same keyword will obtain different weights in different query contexts. This context-aware adaptability is particularly crucial for modern search engines, where semantic precision directly impacts retrieval quality.
(2) Our HRS-MSLC method employs a multi-stage design to achieve funnel-based filtering (coarse-grained high-precision term screening in the first stage + fine-grained term supplementation in the second stage), enabling progressive recall. Furthermore, by incorporating logical combination strategies, it can more precisely capture query intent. Compared to traditional single-stage OR-based recall approaches, this method maintains high recall rates while significantly improving the quality of retrieved results. Additionally, we find that the multi-stage recall strategy demonstrates superior effectiveness compared to the logical combination strategy. This enhanced performance stems from the broader applicability of the multi-stage strategy, as it is effective for most domain queries. In contrast, the logical combination strategy only works when the retrieval results simultaneously contain all the keywords connected by “AND” operators, which limits its wide application.
(3) Although the improvement effect of SE-KIA is smaller than the overall improvement effect of HRS-MSLC, it outperforms both the multi-stage strategy and the logical combination strategy in HRS-MSLC. The performance advantage of SE-KIA stems from its in-depth analysis and innovative integration of offline resources, including user query logs and corpus-level semantic information, to enhance online query processing. In contrast, the multi-stage strategy and the logical combination strategy in HRS-MSLC operate exclusively on real-time queries without leveraging these additional knowledge sources.
(4) The score of the Hit Rate@1 has improved more than that of the Hit Rate@3, which can be attributed to two main factors. The current top 3 accuracy has reached 94.1%, which suggests that the model already achieves relatively high performance in retrieving relevant candidates within the top three positions. At this performance level, further gains become increasingly challenging, as the remaining errors primarily stem from inherent limitations such as annotation noise or ambiguous long-tail queries that lack clear ground-truth answers. Second, and more significantly, the greater top 1 accuracy improvement demonstrates superior capability in retrieving the single most relevant result, which directly translates to improved user experience in real-world applications.

## 7. Conclusions

This research presents significant improvements in information retrieval quality through enhanced retrieval effectiveness in vertical domain search engine inverted index retrieval. Our proposed framework incorporates two key innovations: (1) The SE-KIA method in the query understanding module dynamically optimizes keyword weights by integrating search query logs, corpus data, and semantic entropy theory, enabling more accurate understanding of user intent. (2) The HRS-MSLC approach in the recall module employs multi-granularity word segmentation and multi-queue retrieval while considering logical relationships, effectively balancing recall quantity with result relevance. Experimental evaluations demonstrate that our method achieves substantial improvements in search retrieval effectiveness, specifically for vertical domain search systems. These advancements contribute valuable insights to information retrieval theory while offering practical benefits for search engine optimization.

The current SE-KIA method relies on offline static keyword co-occurrence word graphs, which cannot adapt to temporal semantic shifts or new keyword pairs. This limitation results in the inability to adjust keyword weights in real time when dealing with constantly changing contexts or emerging terms. Future efforts will incorporate online learning or incremental learning mechanisms to enable dynamic semantic entropy calculation, allowing continuous adaptation to evolving corpora.

## Figures and Tables

**Table 1:** Comparison of weight factors in SE-KIA on the Vertical-UserTest datasets.

▵Weight	Hit Rate@1	Hit Rate@3
0	85.6%	87.5%
0.1	88.1%	89.7%
0.2	89.2%	90.7%
0.3	88.6%	90.2%
0.4	87.8%	89.5%

**Table 2:** Comparison of keyword importance analysis methods on the Vertical-UserTest dataset.

Method	Hit Rate@1	Hit Rate@3
Domain-BM25F	78.2%	80.4%
Static weight	85.6%	87.5%
SE-KIA	89.2%	90.7%

**Table 3:** Comparison of recall strategies on the Vertical-UserTest dataset. A checkmark (✓) indicates that the module is used, while its absence denotes that the module is not activated.

Method			Recall Accuracy	
OR-Based + Single-Stage	Multi-Stage	Logical Combination	Hit Rate@1	Hit Rate@3
✓			85.6%	87.5%
✓	✓		88.6%	90.2%
✓		✓	88.1%	89.7%
✓	✓	✓	90.2%	91.6%

**Table 4:** Comparison of different methods on the Vertical-UserTest dataset.

Method	Hit Rate@1	Hit Rate@3
Domain-BM25F	78.2%	80.4%
Static weight	85.6%	87.5%
SE-KIA	89.2%	90.7%
HRS-MSLC	90.2%	91.6%
SE-MSLC(SE-KIA + HRS-MSLC)	92.9%	94.1%

**Table 5:** Comparison of different methods on the TREC-CAR dataset.

Method	MAP
BM25	15.3
Domain-BM25F	20.7
Static weight	22.4
SE-KIA	23.7
HRS-MSLC	23.9
SE-MSLC(SE-KIA + HRS-MSLC)	24.8

## Data Availability

The data presented in this study are available on request from the corresponding author due to privacy restrictions.
